# Crystal structure of 2-(5-bromo-2-hy­droxy­benzyl­idene)-2,3-di­hydro-1*H*-indene-1,3-dione

**DOI:** 10.1107/S2056989015007434

**Published:** 2015-04-22

**Authors:** Joel T. Mague, Shaaban K. Mohamed, Mehmet Akkurt, Antar A. Abdelhamid, Mustafa R. Albayati

**Affiliations:** a Department of Chemistry, Tulane University, New Orleans, LA 70118, USA; b Chemistry and Environmental Division, Manchester Metropolitan University, Manchester M1 5GD, England; c Chemistry Department, Faculty of Science, Minia University, 61519 El-Minia, Egypt; d Department of Physics, Faculty of Sciences, Erciyes University, 38039 Kayseri, Turkey; e Department of Chemistry, Faculty of Science, Sohag University, 82524 Sohag, Egypt; f Kirkuk University, College of Science, Department of Chemistry, Kirkuk, Iraq

**Keywords:** crystal structure, 3-substituted indan-1,3-diones, hydrogen bonding, π–π inter­actions

## Abstract

The title mol­ecule, C_16_H_9_BrO_3_, deviates slightly from planarity. The benzene ring makes a dihedral angle of 1.02 (9)° with the plane defined by the five-membered ring of the indandione moiety. The latter exhibits a minute twist indicated by the dihedral angle of 0.47 (9)° between the planes of the five- and six-membered rings. An intra­molecular C—H⋯O hydrogen bond between the attached benzene ring with one of the indandione carbonyl O atoms stabilizes the mol­ecular conformation. In the crystal, the mol­ecules form dimers across centres of inversion *via* pairwise O—H⋯O hydrogen bonds. The dimers form stacks running parallel to [010] and inter­act through π–π inter­actions between the five-membered ring of one mol­ecule and the six-membered rings of the indandione moiety of an adjacent mol­ecule [centroid-to-centroid distance = 3.5454 (10) Å].

## Related literature   

Indan-1,3-dione and its analogues are synthons for building highly inter­esting compounds with a wide range of applications in both pharmaceutical and industrial chemistry (Kuhn & Rae, 1971 ▸; Junek & Sterk, 1968 ▸; Kunz & Polansky, 1969 ▸; Aldersley *et al.*, 1983 ▸). For chemical reactions and bio-activities of 3-substituted indan-1,3-diones, see: Hochrainer & Wessely (1966 ▸); Zargar & Khan (2015 ▸).
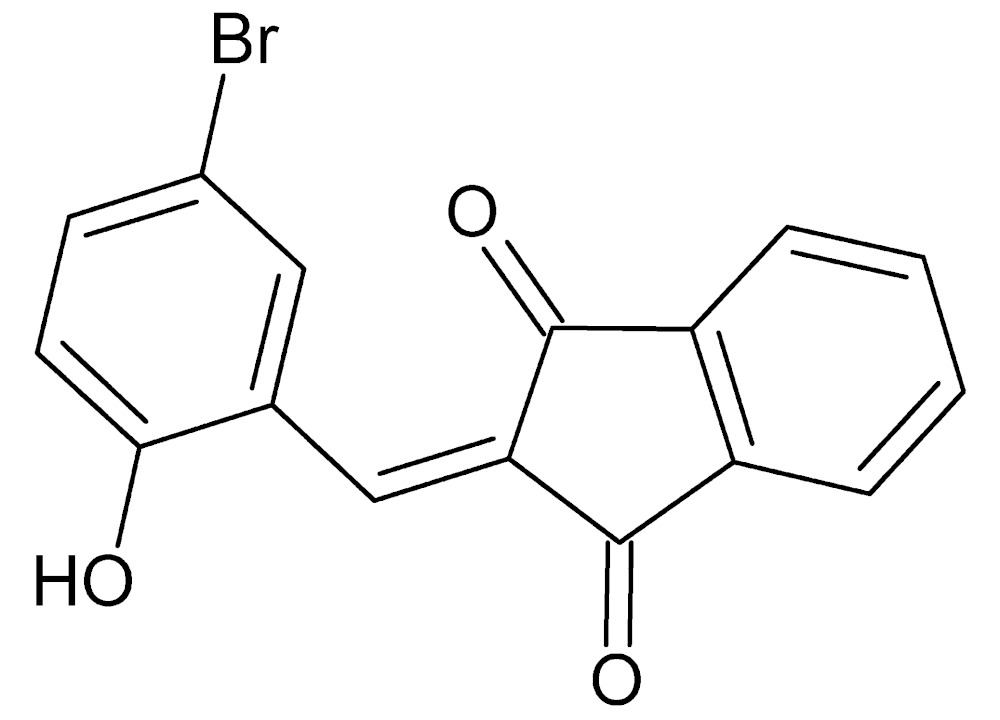


## Experimental   

### Crystal data  

C_16_H_9_BrO_3_
*M* *_r_* = 329.14
Monoclinic, 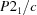

*a* = 13.8820 (4) Å
*b* = 3.8695 (1) Å
*c* = 24.0068 (5) Å
β = 102.483 (1)°
*V* = 1259.07 (6) Å^3^
*Z* = 4
Cu *K*α radiation
μ = 4.50 mm^−1^
*T* = 150 K
0.22 × 0.07 × 0.04 mm

### Data collection  

Bruker D8 VENTURE PHOTON 100 CMOS diffractometer
Absorption correction: numerical (*SADABS*; Bruker, 2014 ▸) *T*_min_ = 0.67, *T*_max_ = 0.84
8943 measured reflections
2510 independent reflections
2386 reflections with *I* > 2σ(*I*)
*R*_int_ = 0.020

### Refinement  

*R*[*F* ^2^ > 2σ(*F* ^2^)] = 0.021
*wR*(*F* ^2^) = 0.056
*S* = 1.09
2510 reflections
181 parameters
H-atom parameters constrained
Δρ_max_ = 0.36 e Å^−3^
Δρ_min_ = −0.25 e Å^−3^

### 

Data collection: *APEX2* (Bruker, 2014 ▸); cell refinement: *SAINT* (Bruker, 2014 ▸); data reduction: *SAINT*; program(s) used to solve structure: *SHELXT* (Sheldrick, 2008 ▸); program(s) used to refine structure: *SHELXL2014* (Sheldrick, 2015 ▸); molecular graphics: *DIAMOND* (Brandenburg & Putz, 2012 ▸); software used to prepare material for publication: *SHELXTL* (Sheldrick, 2008 ▸).

## Supplementary Material

Crystal structure: contains datablock(s) global, I. DOI: 10.1107/S2056989015007434/wm5145sup1.cif

Structure factors: contains datablock(s) I. DOI: 10.1107/S2056989015007434/wm5145Isup2.hkl

**Fig. 1.**
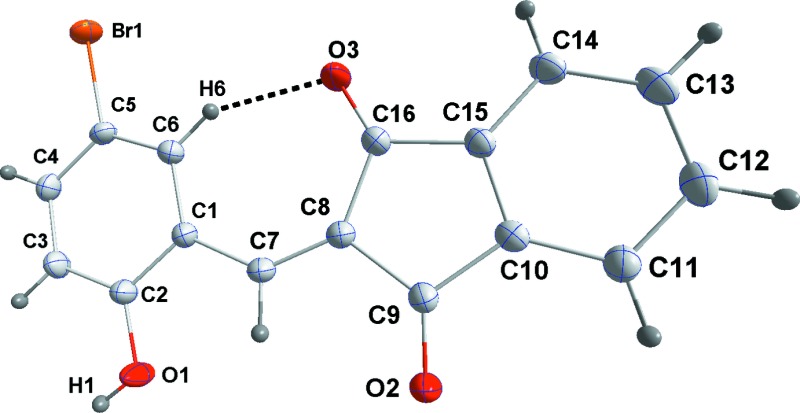
The title molecule with labeling scheme. Displacement ellipsoids are drawn at the 50% probability level. The intramolecular C—H···O interaction is shown as a dotted line.

. DOI: 10.1107/S2056989015007434/wm5145fig1.tif
The title mol­ecule with labeling scheme. Displacement ellipsoids are drawn at the 50% probability level. The intra­molecular C—H⋯O inter­action is shown as a dotted line.

**Fig. 2.**
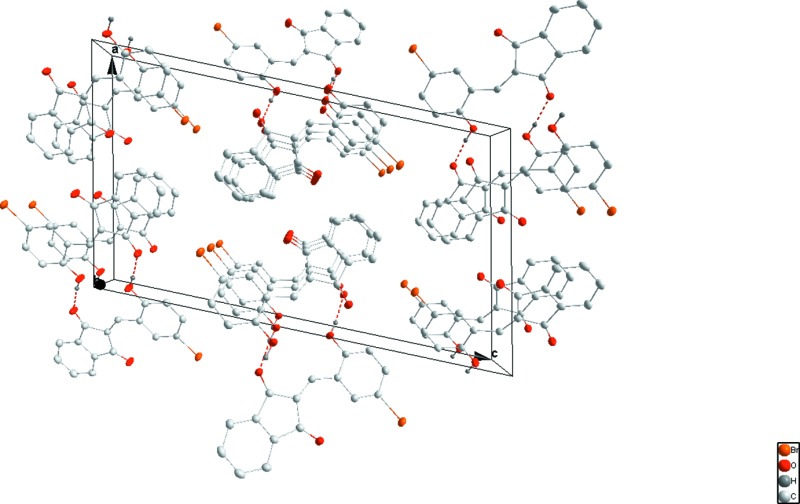
Crystal packing of the title compound viewed down [010]. Intermolecular O—H···O hydrogen bonds are shown as dotted lines.

. DOI: 10.1107/S2056989015007434/wm5145fig2.tif
Crystal packing of the title compound viewed down [010]. Inter­molecular O—H⋯O hydrogen bonds are shown as dotted lines.

CCDC reference: 1059869

Additional supporting information:  crystallographic information; 3D view; checkCIF report

## Figures and Tables

**Table 1:** Hydrogen-bond geometry (, )

*D*H*A*	*D*H	H*A*	*D* *A*	*D*H*A*
C6H6O3	0.95	2.15	2.994(2)	148
O1H1O2^i^	0.84	1.83	2.6641(16)	173

Symmetry code: (i) 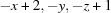.

## supplementary crystallographic information

### S1. Experimental

A mixture of 1 mmol (146 mg) of 1*H*-indene-1,3(2*H*)-dione and 1 mmol (201 mg) of 5-bromo-2-hydroxybenzaldehyde in 30 ml ethanol was refluxed for 30 min. The resulting solid product was collected under vacuum and re-crystallized from ethanol to afford yellow needles suitable for X-ray diffraction in 83% yield.

### S2. Refinement

H-atoms attached to carbon were placed in calculated positions (C—H = 0.95 Å) while that attached to oxygen was placed in a location derived from a difference map and its coordinates adjusted to give a distance O—H = 0.84 Å. All H atoms were included as riding contributions with isotropic displacement parameters 1.2 times those of the attached atoms.

### Crystal data

**Table:**

C_16_H_9_BrO_3_	*F*(000) = 656
*M**_r_* = 329.14	*D*_x_ = 1.736 Mg m^−^^3^
Monoclinic, *P*2_1_/*c*	Cu *K*α radiation, λ = 1.54178 Å
*a* = 13.8820 (4) Å	Cell parameters from 7789 reflections
*b* = 3.8695 (1) Å	θ = 3.3–74.5°
*c* = 24.0068 (5) Å	µ = 4.50 mm^−^^1^
β = 102.483 (1)°	*T* = 150 K
*V* = 1259.07 (6) Å^3^	Needle, yellow
*Z* = 4	0.22 × 0.07 × 0.04 mm

### Data collection

**Table:**

Bruker D8 VENTURE PHOTON 100 CMOS diffractometer	2510 independent reflections
Radiation source: INCOATEC IµS micro–focus source	2386 reflections with *I* > 2σ(*I*)
Mirror monochromator	*R*_int_ = 0.020
Detector resolution: 10.4167 pixels mm^-1^	θ_max_ = 74.5°, θ_min_ = 3.3°
ω scans	*h* = −16→17
Absorption correction: numerical (*SADABS*; Bruker, 2014)	*k* = −4→4
*T*_min_ = 0.67, *T*_max_ = 0.84	*l* = −29→29
8943 measured reflections	

### Refinement

**Table:**

Refinement on *F*^2^	Primary atom site location: structure-invariant direct methods
Least-squares matrix: full	Secondary atom site location: difference Fourier map
*R*[*F*^2^ > 2σ(*F*^2^)] = 0.021	Hydrogen site location: mixed
*wR*(*F*^2^) = 0.056	H-atom parameters constrained
*S* = 1.09	*w* = 1/[σ^2^(*F*_o_^2^) + (0.0301*P*)^2^ + 0.6412*P*] where *P* = (*F*_o_^2^ + 2*F*_c_^2^)/3
2510 reflections	(Δ/σ)_max_ = 0.003
181 parameters	Δρ_max_ = 0.36 e Å^−^^3^
0 restraints	Δρ_min_ = −0.25 e Å^−^^3^

### Special details

**Table:**

Geometry. All e.s.d.'s (except the e.s.d. in the dihedral angle between two l.s. planes) are estimated using the full covariance matrix. The cell e.s.d.'s are taken into account individually in the estimation of e.s.d.'s in distances, angles and torsion angles; correlations between e.s.d.'s in cell parameters are only used when they are defined by crystal symmetry. An approximate (isotropic) treatment of cell e.s.d.'s is used for estimating e.s.d.'s involving l.s. planes.
Refinement. Refinement of *F*^2^ against ALL reflections. The weighted *R*-factor *wR* and goodness of fit *S* are based on *F*^2^, conventional *R*-factors *R* are based on *F*, with *F* set to zero for negative *F*^2^. The threshold expression of *F*^2^ > σ(*F*^2^) is used only for calculating *R*-factors(gt) *etc*. and is not relevant to the choice of reflections for refinement. *R*-factors based on *F*^2^ are statistically about twice as large as those based on *F*, and *R*- factors based on ALL data will be even larger. H-atoms attached to carbon were placed in calculated positions (C—H = 0.95 Å) while that attached to oxygen was placed in a location derived from a difference map and its coordinates adjusted to give O—H = 0.84 Å. All were included as riding contributions with isotropic displacement parameters 1.2 times those of the attached atoms.

### Fractional atomic coordinates and isotropic or equivalent isotropic displacement parameters (Å^2^)

**Table:**

	*x*	*y*	*z*	*U*_iso_*/*U*_eq_	
Br1	0.74761 (2)	0.32460 (5)	0.72665 (2)	0.02597 (8)	
O1	1.00458 (9)	−0.0525 (4)	0.56417 (5)	0.0324 (3)	
H1	1.0561	−0.1618	0.5789	0.039*	
O2	0.82877 (9)	0.4018 (4)	0.39794 (5)	0.0275 (3)	
O3	0.63542 (9)	0.6391 (4)	0.53435 (5)	0.0331 (3)	
C1	0.85867 (12)	0.2074 (4)	0.57969 (6)	0.0188 (3)	
C2	0.94845 (11)	0.0308 (4)	0.60120 (6)	0.0216 (3)	
C3	0.97616 (11)	−0.0550 (4)	0.65901 (6)	0.0227 (3)	
H3	1.0370	−0.1702	0.6731	0.027*	
C4	0.91553 (12)	0.0270 (4)	0.69553 (6)	0.0215 (3)	
H4	0.9339	−0.0334	0.7348	0.026*	
C5	0.82703 (12)	0.1991 (4)	0.67450 (6)	0.0195 (3)	
C6	0.79773 (12)	0.2902 (4)	0.61786 (6)	0.0195 (3)	
H6	0.7370	0.4079	0.6046	0.023*	
C7	0.83772 (12)	0.2944 (4)	0.51956 (6)	0.0196 (3)	
H7	0.8883	0.2221	0.5011	0.024*	
C8	0.76223 (11)	0.4559 (4)	0.48379 (6)	0.0193 (3)	
C9	0.76467 (11)	0.4982 (4)	0.42218 (6)	0.0195 (3)	
C10	0.67288 (11)	0.6780 (4)	0.39402 (7)	0.0191 (3)	
C11	0.64124 (13)	0.7720 (4)	0.33723 (7)	0.0235 (3)	
H11	0.6801	0.7253	0.3100	0.028*	
C12	0.55046 (13)	0.9372 (4)	0.32177 (7)	0.0262 (3)	
H12	0.5268	1.0061	0.2833	0.031*	
C13	0.49331 (12)	1.0039 (5)	0.36193 (7)	0.0269 (3)	
H13	0.4315	1.1172	0.3502	0.032*	
C14	0.52519 (12)	0.9075 (5)	0.41869 (7)	0.0243 (3)	
H14	0.4862	0.9515	0.4459	0.029*	
C15	0.61626 (12)	0.7441 (4)	0.43405 (7)	0.0202 (3)	
C16	0.66754 (12)	0.6133 (4)	0.49114 (7)	0.0216 (3)	

### Atomic displacement parameters (Å^2^)

**Table:**

	*U*^11^	*U*^22^	*U*^33^	*U*^12^	*U*^13^	*U*^23^
Br1	0.03096 (11)	0.02856 (12)	0.02164 (10)	0.00308 (7)	0.01281 (7)	−0.00170 (6)
O1	0.0248 (6)	0.0525 (8)	0.0217 (5)	0.0200 (6)	0.0089 (4)	0.0075 (6)
O2	0.0239 (6)	0.0394 (7)	0.0207 (5)	0.0109 (5)	0.0084 (4)	0.0042 (5)
O3	0.0278 (6)	0.0513 (8)	0.0223 (6)	0.0172 (6)	0.0102 (5)	0.0053 (5)
C1	0.0181 (7)	0.0208 (8)	0.0175 (7)	0.0013 (6)	0.0038 (6)	−0.0007 (6)
C2	0.0193 (7)	0.0260 (8)	0.0200 (7)	0.0031 (7)	0.0057 (6)	0.0000 (6)
C3	0.0193 (7)	0.0266 (8)	0.0208 (7)	0.0037 (7)	0.0012 (6)	0.0023 (6)
C4	0.0236 (7)	0.0225 (8)	0.0172 (6)	−0.0010 (7)	0.0019 (6)	0.0004 (6)
C5	0.0206 (7)	0.0203 (8)	0.0193 (7)	−0.0011 (6)	0.0079 (6)	−0.0030 (6)
C6	0.0186 (7)	0.0201 (8)	0.0199 (7)	0.0023 (6)	0.0042 (6)	−0.0011 (6)
C7	0.0193 (7)	0.0217 (8)	0.0189 (7)	0.0021 (6)	0.0062 (6)	−0.0013 (6)
C8	0.0190 (7)	0.0208 (7)	0.0184 (7)	0.0014 (6)	0.0049 (5)	−0.0006 (6)
C9	0.0190 (7)	0.0201 (7)	0.0189 (7)	0.0011 (6)	0.0032 (5)	0.0006 (6)
C10	0.0181 (7)	0.0173 (7)	0.0215 (7)	−0.0004 (6)	0.0033 (6)	−0.0010 (5)
C11	0.0262 (8)	0.0230 (8)	0.0207 (7)	0.0017 (7)	0.0039 (6)	0.0016 (6)
C12	0.0299 (8)	0.0222 (8)	0.0224 (7)	0.0013 (7)	−0.0033 (6)	0.0022 (6)
C13	0.0218 (8)	0.0230 (8)	0.0317 (8)	0.0043 (7)	−0.0032 (6)	−0.0005 (7)
C14	0.0191 (7)	0.0259 (8)	0.0271 (8)	0.0039 (7)	0.0031 (6)	−0.0019 (7)
C15	0.0181 (7)	0.0203 (7)	0.0211 (7)	0.0010 (6)	0.0019 (6)	−0.0010 (6)
C16	0.0196 (7)	0.0238 (8)	0.0211 (7)	0.0040 (6)	0.0037 (6)	0.0005 (6)

### Geometric parameters (Å, º)

**Table:**

Br1—C5	1.9014 (15)	C7—H7	0.9500
O1—C2	1.3418 (19)	C8—C16	1.493 (2)
O1—H1	0.8400	C8—C9	1.4956 (19)
O2—C9	1.2221 (19)	C9—C10	1.481 (2)
O3—C16	1.218 (2)	C10—C11	1.387 (2)
C1—C6	1.412 (2)	C10—C15	1.390 (2)
C1—C2	1.417 (2)	C11—C12	1.390 (2)
C1—C7	1.449 (2)	C11—H11	0.9500
C2—C3	1.398 (2)	C12—C13	1.399 (3)
C3—C4	1.377 (2)	C12—H12	0.9500
C3—H3	0.9500	C13—C14	1.390 (2)
C4—C5	1.393 (2)	C13—H13	0.9500
C4—H4	0.9500	C14—C15	1.390 (2)
C5—C6	1.378 (2)	C14—H14	0.9500
C6—H6	0.9500	C15—C16	1.490 (2)
C7—C8	1.355 (2)		
			
C2—O1—H1	113.9	O2—C9—C10	124.70 (14)
C6—C1—C2	118.43 (14)	O2—C9—C8	127.73 (14)
C6—C1—C7	125.05 (14)	C10—C9—C8	107.57 (13)
C2—C1—C7	116.51 (14)	C11—C10—C15	121.66 (15)
O1—C2—C3	121.74 (14)	C11—C10—C9	129.07 (15)
O1—C2—C1	117.73 (13)	C15—C10—C9	109.27 (13)
C3—C2—C1	120.53 (14)	C10—C11—C12	117.32 (15)
C4—C3—C2	120.16 (14)	C10—C11—H11	121.3
C4—C3—H3	119.9	C12—C11—H11	121.3
C2—C3—H3	119.9	C11—C12—C13	121.16 (15)
C3—C4—C5	119.47 (14)	C11—C12—H12	119.4
C3—C4—H4	120.3	C13—C12—H12	119.4
C5—C4—H4	120.3	C14—C13—C12	121.23 (15)
C6—C5—C4	121.94 (14)	C14—C13—H13	119.4
C6—C5—Br1	119.65 (12)	C12—C13—H13	119.4
C4—C5—Br1	118.37 (11)	C13—C14—C15	117.44 (15)
C5—C6—C1	119.46 (14)	C13—C14—H14	121.3
C5—C6—H6	120.3	C15—C14—H14	121.3
C1—C6—H6	120.3	C14—C15—C10	121.19 (15)
C8—C7—C1	134.39 (15)	C14—C15—C16	128.67 (15)
C8—C7—H7	112.8	C10—C15—C16	110.15 (14)
C1—C7—H7	112.8	O3—C16—C15	124.43 (15)
C7—C8—C16	133.85 (14)	O3—C16—C8	128.87 (14)
C7—C8—C9	119.83 (14)	C15—C16—C8	106.70 (13)
C16—C8—C9	106.32 (13)		
			
C6—C1—C2—O1	178.65 (15)	C8—C9—C10—C11	−179.35 (16)
C7—C1—C2—O1	−2.4 (2)	O2—C9—C10—C15	179.00 (16)
C6—C1—C2—C3	−0.7 (2)	C8—C9—C10—C15	−0.22 (18)
C7—C1—C2—C3	178.19 (15)	C15—C10—C11—C12	0.3 (2)
O1—C2—C3—C4	−178.38 (17)	C9—C10—C11—C12	179.34 (16)
C1—C2—C3—C4	1.0 (3)	C10—C11—C12—C13	−0.3 (3)
C2—C3—C4—C5	−0.7 (3)	C11—C12—C13—C14	0.0 (3)
C3—C4—C5—C6	0.1 (3)	C12—C13—C14—C15	0.4 (3)
C3—C4—C5—Br1	−177.54 (13)	C13—C14—C15—C10	−0.4 (3)
C4—C5—C6—C1	0.1 (2)	C13—C14—C15—C16	−179.70 (17)
Br1—C5—C6—C1	177.74 (12)	C11—C10—C15—C14	0.1 (3)
C2—C1—C6—C5	0.2 (2)	C9—C10—C15—C14	−179.15 (15)
C7—C1—C6—C5	−178.61 (15)	C11—C10—C15—C16	179.48 (15)
C6—C1—C7—C8	−1.5 (3)	C9—C10—C15—C16	0.27 (19)
C2—C1—C7—C8	179.65 (18)	C14—C15—C16—O3	−1.4 (3)
C1—C7—C8—C16	−0.2 (3)	C10—C15—C16—O3	179.24 (17)
C1—C7—C8—C9	−179.21 (17)	C14—C15—C16—C8	179.14 (17)
C7—C8—C9—O2	0.1 (3)	C10—C15—C16—C8	−0.22 (19)
C16—C8—C9—O2	−179.12 (17)	C7—C8—C16—O3	1.5 (3)
C7—C8—C9—C10	179.33 (15)	C9—C8—C16—O3	−179.35 (18)
C16—C8—C9—C10	0.08 (17)	C7—C8—C16—C15	−179.02 (18)
O2—C9—C10—C11	−0.1 (3)	C9—C8—C16—C15	0.08 (17)

### Hydrogen-bond geometry (Å, º)

**Table:**

*D*—H···*A*	*D*—H	H···*A*	*D*···*A*	*D*—H···*A*
C6—H6···O3	0.95	2.15	2.994 (2)	148
O1—H1···O2^i^	0.84	1.83	2.6641 (16)	173

Symmetry code: (i) −*x*+2, −*y*, −*z*+1.
