# Supplementary figures and images for: Crystal structure of 2-(5-bromo-2-hy­droxy­benzyl­idene)-2,3-di­hydro-1H-indene-1,3-dione
Source: Acta Crystallogr E Crystallogr Commun. 2015 Apr 22;71(Pt 5):o324–5. doi: 10.1107/S2056989015007434 (PMC4420123; doi:10.1107/S2056989015007434)

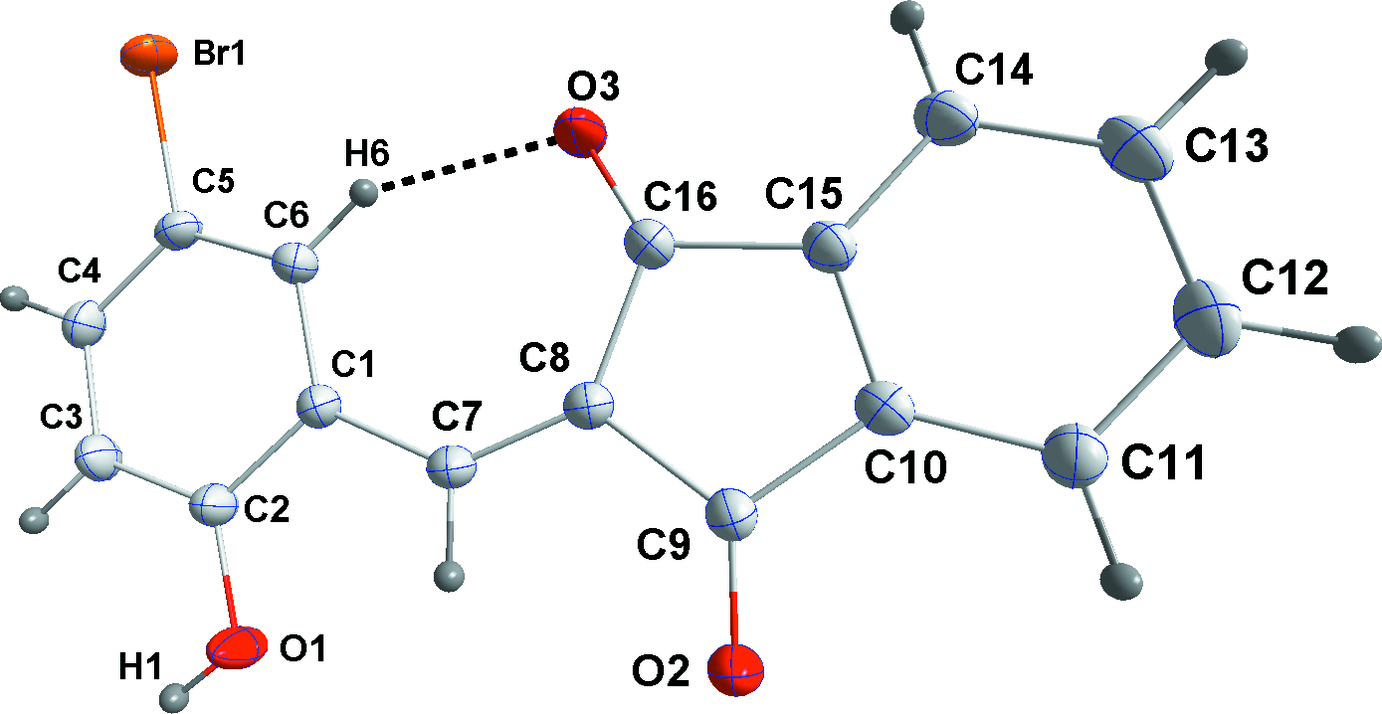

Supplement: Supplementary file 3 [file e-71-0o324-fig1.tif]

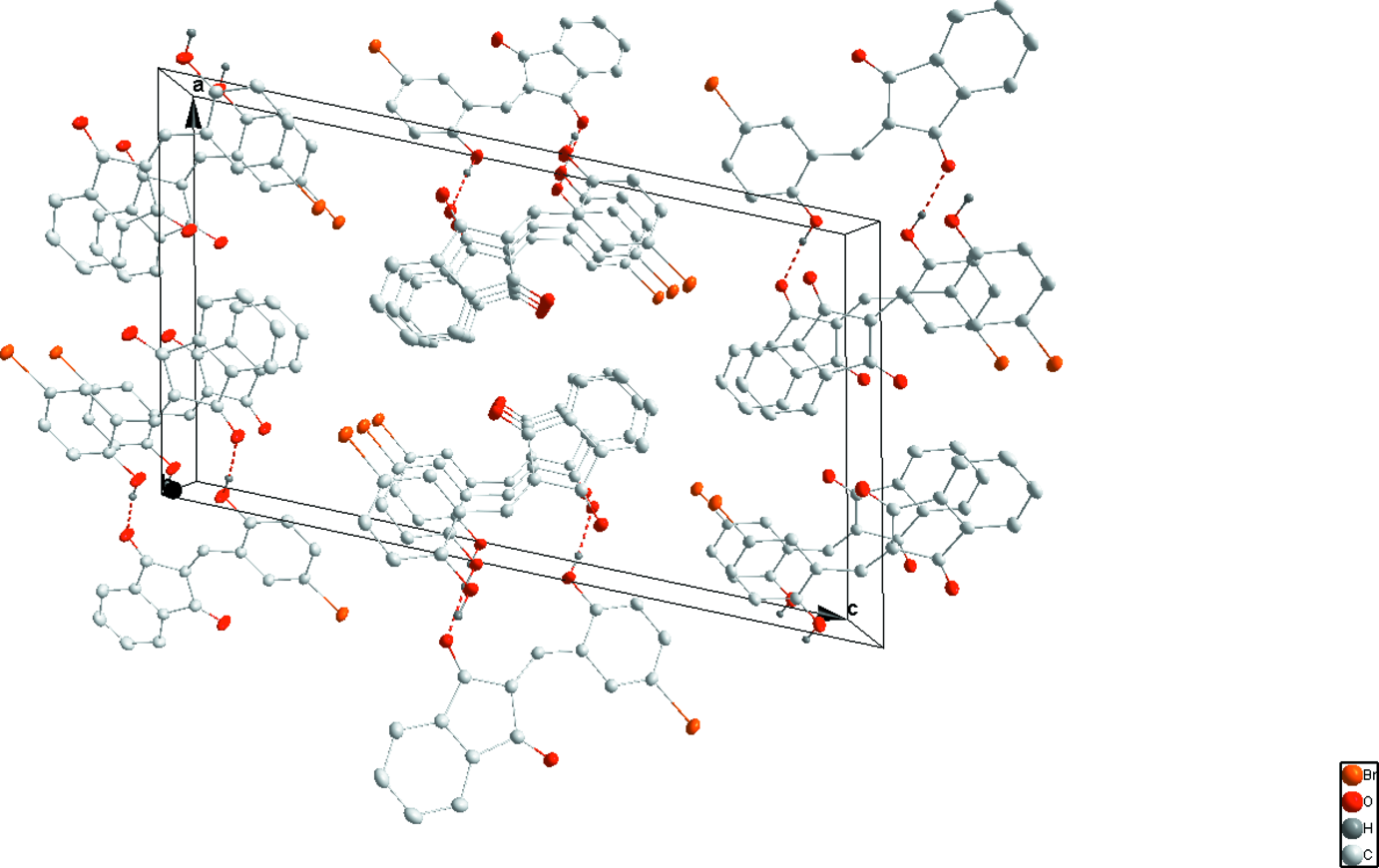

Supplement: Supplementary file 4 [file e-71-0o324-fig2.tif]
